# Vector competence of *Anopheles* and *Culex* mosquitoes for Zika virus

**DOI:** 10.7717/peerj.3096

**Published:** 2017-03-14

**Authors:** Brittany L. Dodson, Jason L. Rasgon

**Affiliations:** 1 Department of Entomology, Pennsylvania State University, University Park, PA, United States; 2 Center for Infectious Disease Dynamics, Pennsylvania State University, University Park, PA, United States; 3 The Huck Institutes of the Life Sciences, Pennsylvania State University, University Park, PA, United States

**Keywords:** Zika virus, Mosquito, Vector competence, Emerging pathogen

## Abstract

Zika virus is a newly emergent mosquito-borne flavivirus that has caused recent large outbreaks in the new world, leading to dramatic increases in serious disease pathology including Guillain-Barre syndrome, newborn microcephaly, and infant brain damage. Although *Aedes* mosquitoes are thought to be the primary mosquito species driving infection, the virus has been isolated from dozens of mosquito species, including *Culex* and *Anopheles* species, and we lack a thorough understanding of which mosquito species to target for vector control. We exposed *Anopheles gambiae*, *Anopheles stephensi*, and *Culex quinquefasciatus* mosquitoes to blood meals supplemented with two Zika virus strains. Mosquito bodies, legs, and saliva were collected five, seven, and 14 days post blood meal and tested for infectious virus by plaque assay. Regardless of titer, virus strain, or timepoint, *Anopheles gambiae*, *Anopheles stephensi*, and *Culex quinquefasciatus* mosquitoes were refractory to Zika virus infection. We conclude that *Anopheles gambiae*, *Anopheles stephensi*, and *Culex quinquefasciatus* mosquitoes likely do not contribute significantly to Zika virus transmission to humans. However, future studies should continue to explore the potential for other novel potential vectors to transmit the virus.

## Introduction

Zika virus (ZIKV) is a mosquito-borne flavivirus first isolated in 1947 from a sentinel rhesus macaque monkey (*Macaca mulatta*), and in 1948 from *Aedes africanus* mosquitoes in Uganda (Dick, Kitchen & Haddow, 1952). Studies have reported ZIKV epizootics in monkeys and sporadic human infections in Africa and Asia (MacNamara, 1954; Olson et al., 1981; McCrae & Kirya, 1982), but recently, ZIKV has spread to other areas of the world including South Pacific islands and, recently, large outbreaks in the Americas (Roth et al., 2014; Guerbois et al., 2016; Tognarelli et al., 2016). Additionally, ZIKV can be directly transmitted between humans (Foy et al., 2011; Peterson et al., 2016), and may cause severe symptoms such as Guillain-Barre syndrome (Oehler et al., 2014) in adults and a suite of developmental abnormalities in newborns and infants now referred to as congenital Zika syndrome (Rasmussen et al., 2016; Schuler-Faccini et al., 2016).

There are no vaccines or specific therapies against ZIKV; therefore, the current approaches to alleviate disease burden are limited to personal protection and mosquito control (Wong, Poon & Wong, 2016). These approaches require that the correct vectors are identified and targeted (Pates & Curtis, 2005). *Aedes aegypti* and *Aedes albopictus* have been implicated as competent vectors of ZIKV (Marcchette, Garcia & Rudnick, 1969; Grard et al., 2014; Aliota et al., 2016a), but there may be other mosquito species that contribute to the transmission cycle. ZIKV has been identified from over 25 species of mosquitoes, including field-collected *Culex* and *Anopheles* species (Diallo et al., 2014; Althouse et al., 2015). To address this knowledge gap, we evaluated the ability for *An. gambiae*, *An. stephensi*, and *Cx. quinquefasciatus* to become infected with and transmit ZIKV after feeding on a viremic blood meal.

## Materials & Methods

### Mosquitoes and Zika virus

Three species of mosquitoes were used for experiments. The *Anopheles gambiae* colony was kindly provided by Janet Teeple (The Pennsylvania State University, University Park, PA, USA) and was originally obtained from The National Institutes of Health (Bethesda, MD). *Anopheles stephensi* (Liston strain) were provided by Johns Hopkins University (Baltimore, MD, USA). The *Cx*. *quinquefasciatus* colony was provided by the Wadsworth Center (Slingerlands, NY, USA) and was initially derived from a colony maintained by Benzon Research (Carlisle, PA, USA).

Mosquitoes were reared and maintained at the Millennium Sciences Complex insectary at 27 °C ± 1 °C, 12:12 h light:dark diurnal cycle at 80% relative humidity in 30 × 30 × 30-cm cages. Mosquitoes were provided with 10% sucrose *ad libitum* for maintenance and fed human blood (Biological Specialty Corporation, Colmar, PA, USA) for reproduction and virus infections. *Anopheles* larvae were fed ground fish flakes (TetrMin, Melle, Germany) while *Culex* larvae were fed a 1:1:1 mixture of bovine liver powder (MP Biomedicals, Solon, OH, USA), koi pellets (TetraPond Koi Vibrance; TetraPond, Prestons, Australia), and rabbit pellets (Kaytee, Chilton, WI, USA).

Two ZIKV strains were used in our experiments. The ZIKV strain MR766 (kindly provided by Dr. Michael Diamond) is the original-type strain of the virus and was isolated from an infected rhesus macaque in 1947 in Uganda, Africa. The ZIKV strain PRVABC59 was isolated from infected human serum in Puerto Rico in 2015 (provided by BEI Resources, Manassas, VA, USA). Both viruses were passed in Vero cells two (MR766) or three times (PRVABC59), and the supernatant stored in aliquots at −70 °C until used for mosquito infections.

### Vector competence for ZIKV

Single use ZIKV aliquots were thawed briefly at 37 °C and were added to whole human blood (Biological Specialty Corporation, Colmar, PA, USA) 1:1 (final blood meal titers are in Table 1). For biological relevance, we chose ZIKV blood meal titers similar to those reported in humans (Aliota et al., 2016b; Aubry et al., 2016). We also chose a high titer to test whether titer could be a barrier to competence for some mosquito species. Adult female mosquitoes were exposed to ZIKV-infected blood 3–5 days post-emergence via a glass feeder jacketed with 37 °C distilled water. Mosquitoes were allowed to feed for approximately 1 h, then partially- and non-bloodfed females were discarded. An aliquot of each blood meal was archived for ZIKV titer verification by plaque assay on Vero cells (ATCC) as described below.

**Table 1:** Summary of Zika virus blood meal titers and mosquito sample sizes.

			Number of mosquitoes tested			
Mosquito species tested	ZIKV strains tested	Titers of ZIKV bloodmeals (log _10_ PFU/mL)	Day 5 post-bloodmeal	Day 7 post-bloodmeal	Day 14 post-bloodmeal	Total
*An. gambiae*	MR766	4.6	11	24	nt	35
	MR766	7.0	15	19	11	45
*An. stephensi*	MR766	4.3	30	30	35	95
	MR766	7.7	30	30	33	93
*Cx. quinquefasciatus*	MR766	7.5	nt	30	29	59
	PRVABC59	7.3	nt	26	30	56

**Notes.**

nt not tested

ZIKV infection, dissemination and transmission was assessed at five, seven, and 14 days post-blood feeding (Aitken, 1977; Dodson, Kramer & Rasgon, 2011; Dodson, Kramer & Rasgon, 2012). Day 5 transmissions were not performed for *Cx. quinquefasciatus* due to lower numbers of fully-bloodfed females obtained for this species. Female mosquitoes were anesthetized with triethylamine (Sigma, St. Louis, MO, USA), legs from each mosquito were removed and placed separately in a 2-mL tube filled with 1 mL mosquito diluent (MD: 20% heat-inactivated fetal bovine serum (FBS) in Dulbecco’s phosphate-buffered saline, 50 µg/mL penicillin/streptomycin, 50 ug/mL gentamicin, and 2.5 µg/mL fungizone) with a single zinc-plated, steel, 4.5 mm BB (Daisy, Rogers, AR, USA). The proboscis of each mosquito was positioned in a tapered capillary tube containing approximately 10 µL of a 1:1 solution of 50% sucrose and FBS to induce salivation. After 30 min, the contents were expelled into 0.3 mL MD, and bodies were placed individually into a 2-mL tube filled with 1 mL MD and a single zinc-plated, steel 4.5 mm BB. Mosquito body, legs, and salivary secretion samples were stored at −70 °C until tested for ZIKV presence. Mosquito bodies and legs were thawed briefly at 37 °C, homogenized for 30 s with TissueLyser (QIAGEN, Hilden, Germany) at 24 cycles/s, followed by centrifugation for 1 min. Mosquito samples were tested for ZIKV infectious particles by plaque assay on Vero cells. For the duration of the plaque assay, mosquito samples were stored on ice or at 4 °C to prevent any possible loss of viral infectivity. All mosquito experiments with infectious ZIKV were completed in the Eva J. Pell ABSL3 Laboratory at Pennsylvania State University according to established biosafety protocols.

### Zika virus plaque assay

We tested for ZIKV infectious particles in blood meals and mosquito samples by using Vero plaque assays (Payne et al., 2006). Vero cells were grown to a confluent monolayer in 6-well plates at 37 °C and 5% CO_2_. Complete media (1X Dulbecco’s modified-essential media, 100 units/mL penicillin-streptomycin, and 10% fetal bovine serum) was removed and monolayers were inoculated with 100 µl of each sample. Plates were incubated at 37 °C with 5% CO_2_ for 2 h and were rocked every 30 min to prevent cells from desiccating. After adsorption, monolayers were overlaid with equal amounts of complete media and 1.2% agarose, and incubated at 37 °C with 5% CO_2_ for three days. After the incubation period, a secondary overlay was applied that consisted of equal amounts of complete media and 1.2% agarose, as well as 1.5% neutral red solution. Plates were incubated again at 37 °C with 5% CO_2_ for 1 day. Then, plates were placed on a white light transilluminator (Hall Productions, San Luis Obispo, CA, USA), each well was assessed by eye as positive or negative for infectious ZIKV particles, and plaques were counted if applicable. Positive and negative controls were included with every assay to confirm that the assay worked as expected and to ensure cell viability (positive: diluted ZIKV-infected blood meal and diluted ZIKV stocks; negative: complete cell culture media and MD).

## Results and Discussion

ZIKV was detected in all plaque assay positive controls and was verified in infectious blood meals provided to all experimental mosquitoes. In total, 80 *An. gambiae*, 188 *An*. *stephensi*, and 115 *Cx*. *quinquefasciatus* were tested for ZIKV. A complete summary of our sample sizes is provided in Table 1. Regardless of titer, timepoint, or virus strain, no samples from *An. gambiae*, *An. stephensi*, or *Cx. quinquefasciatus* were positive for infectious ZIKV particles.

ZIKV incidence and medical complications associated with infection have increased rapidly in the Americas, and the scientific community is attempting to catch up (Waddell & Greig, 2016). There is much we do not yet understand about the details of the ZIKV transmission cycle. Emphasis has been placed on *Aedes* mosquitoes as primary vectors, but there are other species that potentially could contribute to the transmission cycle in important ways (Pujhari & Rasgon, 2016; Waddell & Greig, 2016).

*Anopheles* mosquitoes most notably transmit malaria parasites, but could also be responsible for epidemics of O’Nyong-nyong virus (Haddow, Davies & Walker, 1960; Corbet, Williams & Gillett, 1961; Lutwama et al., 1999), an alphavirus genetically and serologically related to Chikungunya virus (Williams & Woodall, 1961). Importantly, ZIKV has been previously isolated from *An. gambiae* and *An. coustani* in the field (Althouse et al., 2015; Pujhari & Rasgon, 2016; Waddell & Greig, 2016). However, our data suggest that *An. gambiae* and *An*. *stephensi* were refractory to ZIKV infection even at a high titer (>7 logs). Isolation of ZIKV from whole field mosquitoes is insufficient to prove that the mosquito is competent to transmit the virus to a new host. Field mosquitoes may be positive for ZIKV simply because of an infectious, undigested blood meal. Isolation of infectious particles from the tissues as legs, wings or head and from the saliva is necessary to show that the mosquito has the ability to replicate/disseminate and transmit the virus, respectively.

*Culex* mosquitoes are capable of transmitting flaviviruses (Goddard et al., 2002; Mackenzie, Gubler & Petersen, 2004), as well as alphaviruses (Reisen et al., 1990) and bunyaviruses (Turell et al., 1996). Two studies have reported evidence that *Cx*. *quinquefasciatus* mosquitoes may be vectors for ZIKV (Guedes et al., 2016; Guo et al., 2016). However, our data suggest that *Cx*. *quinquefasciatus* are refractory to infection with high titers of two ZIKV strains, the original African-type strain (MR766) and a strain isolated from the recent outbreak in the Americas (PRVABC59). Our data are consistent with results from other groups that were unable to infect laboratory *Cx. pipiens* and recently colonized *Cx. quinquefasciatus* (Aliota et al., 2016a; Amraoui et al., 2016; Boccolini et al., 2016; Hall-Mendelin et al., 2016; Huang et al., 2016; Fernandes et al., 2016; Weger-Lucarelli et al., 2016). These conflicting results may be due to differences in the genetic background of the tested mosquito colonies, the viral strains examined, or different assay methodologies. For instance, in the studies that reported *Culex* as a possible ZIKV vector, virus was detected by PCR of viral RNA in mosquito samples, salivary secretions (Guedes et al., 2016) or tissue from mice infected by mosquito bite (Guo et al., 2016). Many studies have shown that PCR can detect viral RNA that is not infectious (Tabachnick et al., 1996; Aubry et al., 2016; Musso et al., 2016). Therefore, virus identification from mosquitoes or bitten hosts by molecular methods is suggestive, but does not necessarily mean the species is capable of becoming infected with and transmitting infectious virus (Bernard et al., 2001; Kramer & Bernard, 2001). Further experiments assessing viable virus transmission in *Culex* mosquitoes are clearly warranted to fully confirm or refute the vector status of this important genus.

Although our results suggest that *An. gambiae*, *An. stephensi*, and *Cx. quinquefasciatus* do not contribute to ZIKV transmission to humans, we cannot rule out that there are other members of those genera that could serve as vectors. Future studies should continue to explore novel vectors for ZIKV, especially given that species other than *Aedes* have been implicated in the spread of arthropod-borne viruses into novel environments (Venkatesan & Rasgon, 2010).

## Conclusions

In conclusion, our data emphasize the importance of exploring the vector competence of diverse mosquito species for emerging pathogens. Our results suggest that *An. gambiae*, *An. stephensi*, and *Cx. quinquefasciatus* are unable to become infected with ZIKV, even at high titers. We also suggest that studies examining vector competence of mosquitoes for ZIKV and other viral pathogens be performed using infectious virus plaque assays, and that the use of PCR-based detection methods be avoided.

## Additional Information and Declarations

### Data Availability

The following information was supplied regarding data availability: All raw data are included in Table 1.
